# In Vivo Study of Spherical Gold Nanoparticles: Inflammatory Effects and Distribution in Mice

**DOI:** 10.1371/journal.pone.0058208

**Published:** 2013-02-28

**Authors:** Hui Chen, Alisha Dorrigan, Sonia Saad, Dominic J. Hare, Michael B. Cortie, Stella M. Valenzuela

**Affiliations:** 1 School of Medical and Molecular Biosciences, Faculty of Science, University of Technology Sydney, Sydney, New South Wales, Australia; 2 Institute for Nanoscale Technology, University of Technology Sydney, Sydney, New South Wales, Australia; 3 Renal Research Group, Kolling Institute of Medical Research, Sydney University, Sydney, New South Wales, Australia; 4 Elemental Bio-imaging Facility, University of Technology Sydney, Sydney, New South Wales, Australia; Tohoku University, Japan

## Abstract

**Objectives:**

Gold nanoparticles (AuNPs) of 21 nm have been previously well characterized *in vitro* for their capacity to target macrophages via active uptake. However, the short-term impact of such AuNPs on physiological systems, in particular resident macrophages located in fat tissue *in vivo*, is largely unknown. This project investigated the distribution, organ toxicity and changes in inflammatory cytokines within the adipose tissue after mice were exposed to AuNPs.

**Methods:**

Male C57BL/6 mice were injected intraperitoneally (IP) with a single dose of AuNPs (7.85 μg AuNPs/g). Body weight and energy intake were recorded daily. Tissues were collected at 1 h, 24 h and 72 h post-injection to test for organ toxicity. AuNP distribution was examined using electron microscopy. Proinflammatory cytokine expression and macrophage number within the abdominal fat pad were determined using real-time PCR.

**Results:**

At 72 hours post AuNP injection, daily energy intake and body weight were found to be similar between Control and AuNP treated mice. However, fat mass was significantly smaller in AuNP-treated mice. Following IP injection, AuNPs rapidly accumulated within the abdominal fat tissue and some were seen in the liver. A reduction in TNFα and IL-6 mRNA levels in the fat were observed from 1 h to 72 h post AuNP injection, with no observable changes in macrophage number. There was no detectable toxicity to vital organs (liver and kidney).

**Conclusion:**

Our 21 nm spherical AuNPs caused no measurable organ or cell toxicity in mice, but were correlated with significant fat loss and inhibition of inflammatory effects. With the growing incidence of obesity and obesity-related diseases, our findings offer a new avenue for the potential development of gold nanoparticles as a therapeutic agent in the treatment of such disorders.

## Introduction

Biomedical applications of gold nanoparticles (AuNPs) are rapidly increasing due to their attractive properties of relatively low cytotoxicity, a high capacity to target cells, readily functionalized surfaces and a tunable optical absorption peak. Gold compounds such as gold sodium thiomalate have long been used to treat some forms of arthritis [1], [2] but the gold in these substances is in the more active oxidized form whereas in nanoparticles it is present as the comparatively inert metallic form. Nowadays, AuNPs are used effectively in laboratory-based *in vivo* research either as a diagnostic imaging agent or as a therapeutic agent in experimental gene and drug delivery and photothermal therapeutics [3]–[5]. AuNP-based delivery systems are being widely explored for use in cancer chemotherapy treatment as they offer increased drug efficacy with low toxicity to healthy tissue, high biocompatibility, along with versatile production methods, which enable custom design [1]. At least one product of this type is now in clinical trials [6]. AuNPs have thus been predicted to have a promising future in mainstream clinical practice [3], [4]. Although AuNPs can be synthesized in a range of morphologies, most current activity is focused on exploiting either nanospheres and nanorods.

AuNPs with sizes in the 1–100 nm range attain useful optical, catalytic and electronic properties which are significantly different from those of bulk gold [5], [7]. The diverse characteristics and applications of AuNPs are due to their facile production into different sizes and shapes, as well as ease of surface modification. Size and shape are critical factors that significantly influence their cellular interactions [8], [9], and in turn their applications. For example, uptake of spherical AuNPs by cells was found to be considerably easier compared to rod-shaped AuNPs. Spherical AuNPs between 20–30 nm in diameter are capable of targeting cells through active and passive means [10], [11], which is likely to induce physiological changes following the cellular interaction between the AuNPs and target cells *in vivo*. Naked AuNPs (uncoated but still possessing a protective electrostatically-adsorbed layer of ions such as citrate) have been shown to have a higher rate of cellular uptake compared to conjugated ones, due to the adsorption of serum proteins onto their surface; while conjugation with materials such as polyethylene glycol can reduce cell surface interactions [12]. Therefore, uncoated spherical AuNPs of 21 nm became the focus of this study.

Cellular uptake of AuNPs can occur via endocytosis or phagocytosis. Macrophage cells are specifically adept at the phagocytosis of particulate material. A primary function of the macrophage is to remove dead cell material in chronic inflammation. Macrophages can migrate into tissues where they develop into resident macrophages, such as those found in fat tissues known as adipose tissue macrophages (ATMs). Local macrophages within the connective tissue ingest foreign materials such as pathogens and recruit additional macrophages if needed. These local macrophages also produce cytokines including TNFα and IL-6, that affect the systemic inflammatory state [13], [14]. As such, changing local macrophage behavior may affect the progress of inflammation-related disorders. These characteristics of macrophages make them a suitable target of AuNPs, in particular, for naked AuNPs. Upon uptake of the AuNPs, macrophages may demonstrate unwanted immune responses due to the foreign objective nature. However, some studies suggest that AuNPs alone do not initiate inflammatory responses within macrophages [15].

The question of the toxicity or otherwise of naked AuNPs is quite controversial [16]. Numerous studies report no adverse biological effect in either *in vitro* or *in vivo* experiments [9], [17]–[23] and it has been pointed out that some observed effects are due to substances conjugated to or adsorbed on the AuNPs, rather than to the AuNPs themselves [17], [18], [21], [24]. In the case of exceedingly small clusters (sub-3 nm) the effects of core and stabilizing ligand shell cannot be readily separated anyway and it is generally agreed that these composite entities have considerably more reactive (and therefore potentially more toxic) chemical properties overall than particles of greater than 5 nm in size [16], [17], [25]. However, for most studies and applications, the real question is whether nanoparticles in the 5 to 100 nm size range have any effect on cells. Dosage is obviously also a factor [26]. As mentioned, most studies are negative, but there are some reports of naked AuNPs exerting a measureable influence on some aspect of cellular activity. Under certain circumstances naked AuNPs have been reported to be cytotoxic [27], down-regulate or up-regulate inflammatory and other functions [18], [28], [29], suppress cell proliferation [30], [31] including angiogenesis [32], reduce the level of reactive oxygen species (ROS) [18], bind to or interfere with DNA [33]–[35], act as an effective adjuvant [36] or cause changes in cell morphology [30], [31]. In addition, it is highly probable that any naked AuNPs in a living organism will be rapidly coated with host proteins [37]. In contrast, coating the AuNP with PEG acts to prevent this [38].

Drawing conclusions from these previous findings is limited, as each study has used different sized gold nanoparticles, with various coatings and shapes, and the results are seemingly contradictory in many instances [39]. As size and shape largely appear to determine the *in vivo* activity of AuNPs, further studies are needed which focus on *in vivo* interactions and safety of individual types of AuNPs synthesized using different methods. Here we investigate the tissue distribution and toxicity caused by a post-single intraperitoneal injection of 21 nm spherical “citrate” AuNPs in mice, as well as their short-term impact on the physiological system of the mice.

## Materials and Methods

### 1. AuNP Synthesis and Endotoxin Screening

The AuNPs were prepared in Milli-Q water as previously described [10]. The shape and size of the AuNPs were confirmed using scanning electron microscopy (SEM). The particles were confirmed to be spherical in shape, with an average diameter of 21.3±0.7 nm. The AuNP/water suspension was autoclaved prior to use.

Three aliquots of the AuNP suspension were screened in duplicate for the presence of endotoxin using a commercially available chromogenic Limulus Amebocyte Lysate Endotoxin Assay Kit (Genscript, NJ, USA) as per the manufacturer's protocol.

### 2. In vivo Mouse Studies

#### 2.1 Administration and Dosage of the AuNPs

Male C57Bl/6 mice aged 8 weeks (Animal Resource Centre, WA, Australia) were housed at 20±2°C on a 12∶12 hours light/dark cycle (Lights on at 0600 hours) with 4 mice per cage. Mice were fed standard rodent chow (Gordon's Specialty Stockfeeds, NSW, Australia), with *ad libitum* to water. All procedures were approved by the Animal Care and Ethics Committee at the University of Technology, Sydney (ACEC# 2009-349A).

All administered solutions were sterile prior to injection. At time 0, three groups of mice were injected intraperitoneally (i.p.). The first group was injected with a single dose of AuNPs made up to a final volume of 0.2 mL water (7.85 μg AuNPs/g body weight, AuNP group). This dose was modified from a previous publication [40]. The second group was injected with 0.2 mL Milli-Q water each (Control group). A third group was injected with a single dose of saline (0.2 mL/mouse, Saline group). This group was included as the internal control for the Control group treated with Milli-Q water to determine the safety of water-only injection. Mice were then sacrificed from each group following treatment at 1 h, 24 h, and 72 h. As previous studies have suggested an anorexigenic effect of AuNPs [27], food intake was measured daily. Pre-weighed rodent chow was placed on the cage lid and collected after 24 h including any pellets found on the cage bedding. The difference was divided by 4 to give the daily food intake per mouse. Body weight was monitored daily for mice sacrificed at 24 h and 72 h.

#### 2.2 Mouse Tissue collection

Tissues were harvested from the mice at 1 h, 24 h and 72 h post-injection. At the conclusion of each time-point the mice were euthanized using Pentothal (0.1 mg/g, i.p., Abbott Diagnostics, NSW, Australia). Blood was collected via cardiac puncture and blood glucose was measured using a hand-held glucose meter (Accu-Check®, Roche, CA, USA). Blood was then centrifuged (13,000 g for 5 min) and plasma was stored at −80°C for further analysis. The mice were sacrificed by decapitation. Brain, heart, spleen, kidneys and liver, along with the abdominal fat were collected. Fat pads and organs were fixed in 10% formalin and stored in 70% ethanol, followed by paraffin embedding. Some abdominal fat was snap frozen in liquid nitrogen and then stored at −80°C.

#### 2.3 Scanning Electron Microscopy (SEM) and Laser Ablation Inductively Coupled Plasma Mass Spectrometry (LA-ICP-MS scanning)

Paraffin embedded tissues from the control and AuNP groups were sliced into 15 μm thick sections, and mounted on a silicon (conductive) substrate for subsequent SEM imaging. All SEM imaging was conducted on the LEO Supra 55VP (Zeiss) SEM using backscatter mode. The SEM was set to variable pressure in high current at 20 kV using a 120 μm aperture. To ensure that the bright spherical structures were in fact gold, X-ray acquisition of atomic elements was performed using INCA analysis software.

Snap-frozen abdominal fat tissue were cryosectioned into 15 μm thick frozen sections and mounted on glass slides for subsequent LA-ICP-MS analysis. The method used employed a New Wave Research UP213 laser ablation unit (Kenelec Technologies, Victoria, Australia) coupled to an Agilent Technologies 7500 cx inductively coupled plasma-mass spectrometer (Agilent Technologies Australia, Victoria, Australia) was used for the preparation of laser ablation imaging. The UP213 laser unit employed a Nd: YAG solid-state laser source emitting a 213 nm laser pulse in the fifth harmonic. A Large Format Cell (LFC) was fitted to the UP213 unit, which was connected to the ICP-MS via polyvinylchloride tubing. The 7500 cx ICP-MS was fitted with a ‘cs’ lens system for enhanced sensitivity. The LA-ICP-MS system was tuned for sensitivity prior to each experiment using NIST 612 Trace Elements in Glass. Each line of ablation produced a single data file. Images were produced by reducing each data file into a single exportable format for imaging software suites. Measured masses included *m*/*z* 13 (for signal normalization) and *m*/*z* 197. The method used for image reduction used Interactive Spectral Imaging Data Analysis Software (ISIDAS), an in-house package written in the Python programming language. The processed data was exported as a. vtk file readable by MayaVi2 (Enthought, Inc., TX, USA). The colour indicates the strength of the signal which is received following transport of an ablated tissue section to the ICPMS, and is directly related to the concentration of the AuNPs [41].

#### 2.4 Urine tests and kidney morphological studies

Urine was collected from the bladder of the sacrificed mice and the presence of protein and blood cells was screened using Multistix® urine analysis strips (Siemens, NY, USA).

Paraffin-embedded kidneys from all 3 groups were sliced into 3 ìm-thick sections. Sections were processed for hematoxylin and eosin (H&E) staining and periodic acid-Schiff (PAS) staining according to conventional procedures [42]. Morphology of all tissue sections was assessed by a single observer in a blinded manner. The glomerular cell numbers stained with H&E was calculated. Ten different glomeruli in the peripheral cortex of each of the seven sections of each group were randomly selected and were examined under a light microscope at a magnification of ×40. The nuclei of all the cells in all groups were counted. PAS was used to highlight basement membranes of glomerular capillary loops and tubular epithelium.

#### 2.5 Plasma Alanine Transaminase (ALT) measurement

Plasma ALT is used as an indicator of liver function, which is increased when liver cell damage presents. Plasma ALT concentration was measured in the Control and AuNP groups using a commercial kit (Cayman Chemical Company, Michigan, USA) following the manufacturer's instructions.

#### 2.6 Quantitative real-time PCR

Total RNA was isolated from the abdominal fat tissue from the Control and AuNP groups using TRIZOL reagent (Invitrogen Australia Pty Ltd, VIC, Australia) according to the manufacturer's instructions. The purified total RNA was used as a template to generate first-strand cDNA synthesis using M-MLV Reverse Transcriptase, RNase H Minus, Point Mutant Kit (Promega, Madison, WI, USA). Applied Biosystem TaqMan® probe/primers (Foster City, CA, USA) that were pre-optimized and validated were used for the quantitative real-time PCR (Eppendorf Realplex^2^, Hamburg, Germany). The target gene probes were labelled with FAM and the housekeeping gene 18 s rRNA was labelled with VIC. Thus gene expression was quantified in a single multiplexing reaction, where the genes of interest were standardized against 18 s rRNA. The average value of the Control group was assigned as the calibrator, against which all other samples are expressed as a fold difference. Fat tissue at 72 h was used in the Control on the consumption that all the marks were stable over the time.

#### 2.7 Statistics

The results were expressed as mean ± standard error (SE). The differences among Control, AuNP and Saline groups were analysed using one-way ANOVA, followed by *post hoc* Fisher's Least Significance Difference tests (Statistica 10. StatSoft Inc. OK, USA). The difference on retroperitoneal fat mass between Control and AuNP groups was selectively analysed using the *student t* test.

## Results

### 1. Endotoxin screening of the AuNPs

The endotoxin screening revealed that there was no detectable level of endotoxin contamination present within the AuNP solutions, with absorbance readings falling below detectable levels (<0.005 EU/mL).

### 2. Food intake, organ mass and functional changes

Compared with the Saline treated mice, the Control group with the Milli-Q water only injection showed no significant changes in the parameters listed in Table 1 except for slightly smaller liver, suggesting no adverse effects were caused by the water-only injection.

**Table 1:** Comparison of various measurements taken from the Control, AuNP and Saline groups.

	Control (n = 8–12)	AuNP (n = 8)	Saline (n = 8)
Body weight start (g)	23.0±0.3	23.1±0.5	22.4±0.3
Body weight 24h (g)	23.2±0.3	23.1±0.5	22.7±0.4
Body weight 72h (g)	23.0±0.3	22.8±0.6	22.8±0.4
Energy intake (g/d)	3.66±0.09	3.53±0.10	3.61±0.19
Blood glucose 72h (mM)	12.0±0.5	11.7±0.6	12.8±0.5
Liver 72h (mg)	1061±37	1041±57	1104±34*
Heart 72h (mg)	118±5	114±7	118±4
Kidney 72h (mg)	170±13	132±7*	141±4
Retroperitoneal fat 72h (mg)	69.6±8.5	46.0±9.3*^t^*	84±12
Mesenteric fat 72h (mg)	316±25	237±31^#^	357±18

Data are expressed as mean ± SE. * P<0.05, compared with Control. ^#^ P<0.05, compared with Control and Saline.

*t* P<0.05, Control vs. AuNP, selective t test.

The body weight and energy intake were similar between the AuNP and Control groups over the 72 h (Table 1). There was no difference in body weight and organ mass at 1 h and 72 h among groups (data not shown). Blood glucose levels, as well as liver and heart weights, were also similar between the Control and AuNP groups. However, mice displayed significantly lower fat mass at 72 h following the single dose injection of AuNPs (P<0.05, Table). The situation with regard to kidney mass was less clear-cut; the AuNP and Saline groups had similar lower mass, compared to that of the Control group.

Urinary screening showed that there was no presence of red blood cells or protein in the urine samples from both the Control and AuNP group at any time point. Negative results were also observed in the Saline group. The kidneys from both the Control and the AuNP-treated mice demonstrated normal structures, as did the Saline injected mice (images not shown). The capillary loops of the glomerulus are well-defined and thin. The endothelial cells were seen in the capillary loops. The mesangial regions were of normal size. Podocytes were present and forming the visceral epithelial surface. Bowman's space was seen along with parietal epithelial cells. In addition, plasma ALT levels were found to be similar between the Control (117±33 ìU/ml) and AuNP (116±25 ìU/ml) mice at 72 h.

### 3. AuNPs tissue distribution

**Figure 1:**
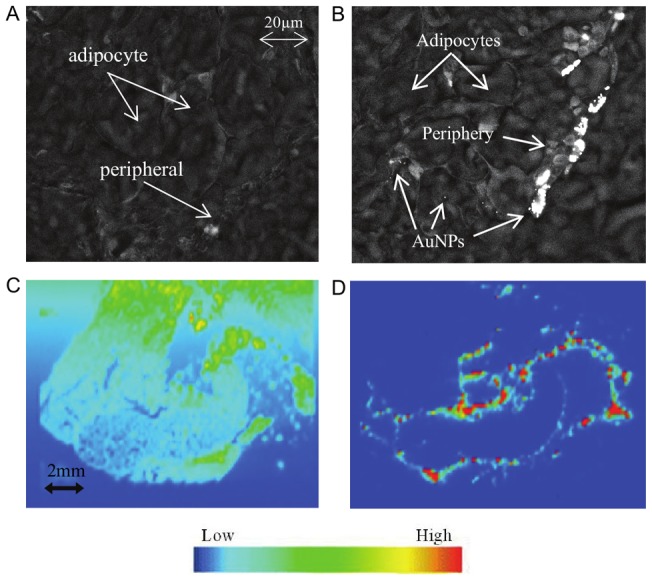
Abdominal adipose tissue distribution of AuNPs following intraperitoneal injection into mice. Scanning electron microscopy (SEM) images of abdominal adipose tissue in (A) control mouse and (B) AuNP-injected mouse at 24 h (Mag 2.01K x). (C, D) Laser ablation inductively coupled plasma mass spectrometry (LA-ICP-MS) images of abdominal adipose tissue at 24 h. (C) displaying the carbon present throughout the tissue sample, of medium to low signal intensity, (D) displays the gold present throughout the tissue sample, concentrated around the periphery of a portion of tissue within the section, of medium to high intensity. The strength of the signal transmitted for a selected element is represented by a corresponding colour, blue signifying the lowest signal strength and red the highest.

From the SEM images, AuNPs can be clearly identified from the dark background of fat tissue cells (Figure 1B), as the AuNPs appear bright white in contrast to the tissue, which was not seen in the control mice (Figure 1A). In the images, AuNPs appear as spheres and clusters in the tissue. SEM imaging revealed that in the abdominal fat, AuNPs were located predominantly within the periphery of the fat tissue, few clusters were seen further into the tissue between the adipocytes at 24 h post AuNPs-injection (Figure 1B). No AuNP-like substance was observed within the tissue of control mice. The presence of AuNPs within tissues was further confirmed by using LA-ICPMS. This was done using a section of abdominal fat from an AuNP injected mouse, at 24 h post-injection (Figure 1C, D). Figure 1C showed the carbon present throughout the fat tissue, suggesting the organic nature of the tissue. The colour spectrum representing varying signal strength is shown in Figure 1D, with yellow to red indicating the presence of gold. In Figure 1D, it is clearly evident that the accumulation of AuNPs in the periphery of the fat tissue, which represents a similar pattern to that observed in the SEM image (Figure 1B). The intensity of the signal in this region indicates that the amount of AuNPs present is substantial. SEM scanning showed that AuNPs were only found in the abdominal fat tissues and the liver. There were no AuNPs present in the other major organs investigated in this study, including brain, kidney and heart (images not shown).

**Figure 2:**
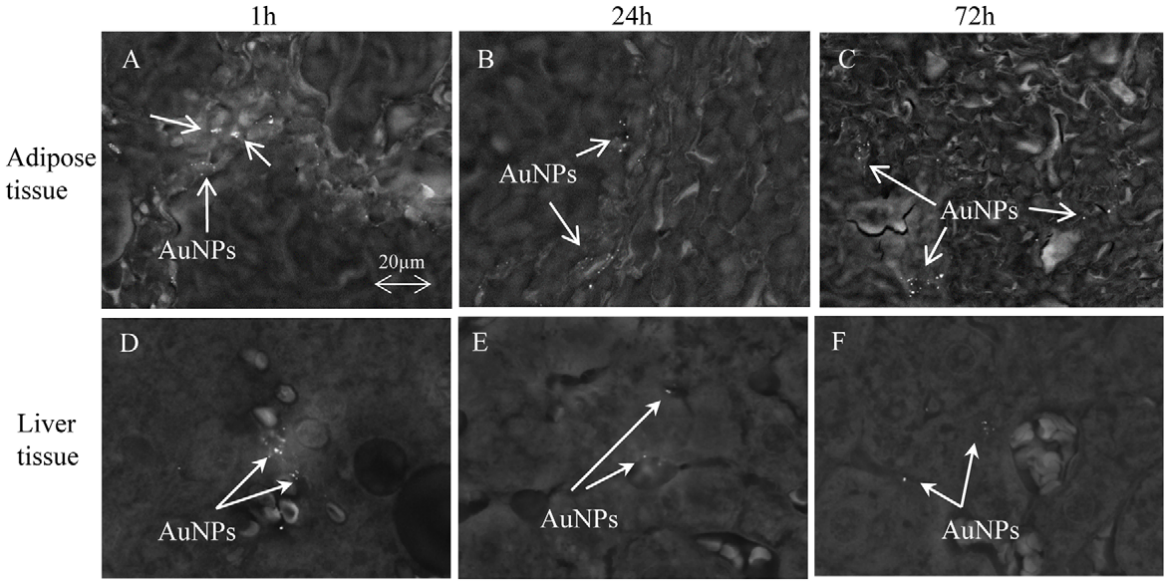
Localisation of AuNPs within specific mouse tissues following IP injection. Scanning electron microscopy (SEM) images of AuNPs in adipose tissue (A, B, C) and liver sections (D, E, F) at 1 h (A, D), 24 h (B, E), and 72 h (C, F) post AuNP injection (Mag 2.01K x).

A time-dependent pattern of AuNP distribution was noted within the abdominal fat and liver. AuNPs were located predominantly at the edge of the fat tissue (Figure 1B), with few clusters seen deep into the tissue between the adipocytes, at 1 h and 24 h (Figure 2A, B). At 72 h, AuNPs were seen to migrate further into the adipose tissue. They tended to accumulate within the connective tissue between adipocytes (Figure 2C). AuNPs on the outside edges of the tissue was visibly reduced. Although the location of the AuNPs within the adipose tissue appeared to change with time, there was however, no noticeable decrease in number of AuNP over the 72 h period.

**Figure 3:**
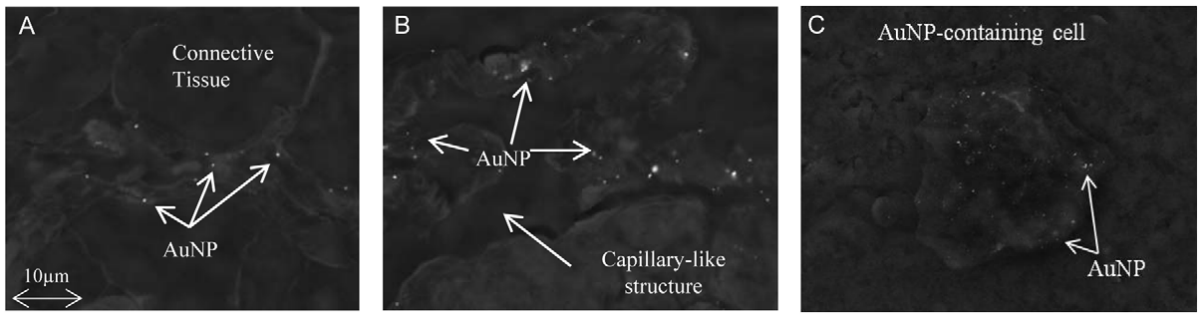
AuNP distribution within mouse liver following IP injection. AuNPs in (A) the liver connective tissue and (B) a 5 µm capillary within the liver adipose tissue (Mag 5.00K x), and in (C) a Kupffer cell within the sinusoid of the liver (Mag 4.19K x).

In the liver, AuNPs were seen to be situated in and around the blood vessels (Figure 2D, E, F), suggesting that they had entered the vascular system and been transported to the liver via the blood circulation. At 72h, AuNPs were seen further away from sinusoids, being identified in and around hepatocytes. The AuNPs also appear to have been phagocytosed by Kupffer cells within the sinusoid as seen in Figure 3D, where a discrete cell body was visualised containing copious numbers of AuNP clusters.

From a higher resolution image, it was noted that the AuNPs mainly distributed in the connective tissues (Figure 3A) and the areas around and within capillary blood vessels (Figure 3B). In the capillary vessel, AuNPs accumulated in what are believed to be macrophage or equivalent local cell types (Figure 3C).

### 4. Changes in cytokine mRNA expression in the adipose tissue following AuNP treatment

**Figure 4:**
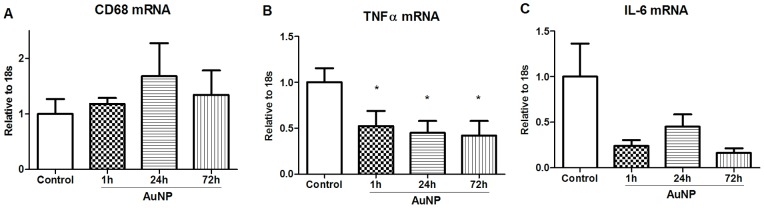
Changes in mRNA cytokine expression levels in mouse abdominal fat tissue at different time points post IP injection of AuNPs. mRNA levels of (A) CD68, (B) TNFα and (C) IL-6 in abdominal fat at different time points post AuNPs injection. Results are expressed as mean ± SE, n = 4–8 in each group. *P<0.05, compared with Control group. CON, control; Au, AuNPs. The differences among groups were analysed using one-way ANOVA, followed by *post hoc* Fisher's Least Significance Difference tests.

The mRNA expression of the marker for macrophage cells, CD68, was not significantly changed over the 3 day period post-AuNP injection compared with the Control group (Figure 4A). However, TNFα mRNA expression was significantly reduced at the 1 h time point, which persisted until 3 days post AuNP injection (Figure 4B). Although IL-6 mRNA expression was also markedly down-regulated over the course of the experiment, it did not reach statistical significance (Figure 4C).

## Discussion

The major finding of the current study is that the chosen dose of spherical AuNPs of 21 nm was safe to all vital organs in the treated mice over the 72 h period of this study. Surprisingly however, there was a significant loss of fat in the AuNP-treated mice without a concomitant significant decrease in their total body weight and energy intake. An apparent inhibition of proinflammatory cytokine expression within the abdominal fat tissue by this type of AnNP was observed *in vivo*. This is an intriguing and unexpected effect of administering the AuNPs.

Gold nanoparticles can be manufactured into a variety of shapes including gold nanospheres, nanorods, nanobelts, nanocages, nanoprisms, and nanostars [43]. The chemical, optical, and electromagnetic properties of gold nanoparticles are strongly influenced by their size and shape. Safety is the first concern to ensure the further application of any new kind of AuNPs *in vivo* does not lead to unwanted side effects.

It has been suggested that particles 1–2 nm in diameter are normally toxic; whereas larger >15 nm gold particles are comparatively nontoxic regardless the cell type tested [25]. Controversially, naked gold nanospheres sized 17 and 37 nm have on occasion been shown to induce anorexia, weight loss, change in fur colour, and liver damage [27]. However, in the current study, naked spherical AuNPs of 21 nm did not cause significant changes in energy intake and body weight within a 72 h period. This could be due to the fact that this size AuNPs did not cross the blood brain barrier as was observed by our SEM imaging; therefore this may have been a reason for a reduced direct detrimental impact on the central nervous system that could potentially lead to anorexia and weight loss. Interestingly, fat mass was found to be significantly reduced in the AuNPs treated mice at 72 h without an overall change to body weight. It seems that this effect could be a novel *in vivo* effect of the unmodified spherical AuNPs of 21 nm, not a result of toxicity. This effect is likely to be size and shape dependent. This novel fat loss effect might lead to a potential application in fat loss related intervention.

This fat loss effect of the AuNPs may be related to their inhibition of pro-inflammatory cytokine expression within the fat tissue, which is predominantly produced by the ATMs [44]. As members of the immune system, ATMs are capable of recognizing, engulfing and removing foreign materials in order to protect the host from disease. Therefore, phagocytosis can be the mode through which AuNPs enter the ATMs, which then results in their functional modulation. During the non-inflammatory state, a large proportion of proinflammatory cytokines, such as TNFα and IL-6, are sourced from abdominal fat tissue. During high fat diet consumption, ATM production of TNFα and IL-6 is closely related to the onset of insulin resistance and glucose intolerance [45], [46]. Thus, ATMs contribute to both local and systemic inflammation and modulate metabolic phenotypes [14]. It has been shown previously that silver nanoparticles and quantum dots could induce the expression of inflammatory mediators in the macrophages upon uptake independent of size [47]. Remarkably, in this study a significant down-regulation of fat TNFα and IL-6 mRNA expression was observed by the presence of AuNPs. This inhibitory effect is rapid and sustained following AuNPs administration. However, blood glucose levels were not different between groups in the short term. Follow-up studies are needed to examine cytokine release from individual cell types (ATM and adipocytes) within the fat tissue *in vitro* and long term plasma cytokine chance *in vivo* after repeated AuNP administration.

CD68 mRNA expression can be used as a marker of ATM number [14]. In our current study, CD68 was unchanged following treatment with AuNPs, suggesting that the reduction in expression of TNFα and IL-6 was due to change in ATM activity rather than number. The inhibition of ATM activity has been shown to increase lipolysis of the fat cells, thus reducing fat mass in obese mice [14], [48]. As such, ATMs have been considered as a promising target for reducing fat mass in obesity. Previous *in vitro* studies have shown that AuNPs conjugated to either peptide or plasma protein, could induce pro-inflammatory cytokine release from macrophages [49], [50], which highlights the importance of size and surface modification on the cellular behaviour of AuNPs. Nevertheless, the inhibition of the inflammatory cytokine expression exerted by our 21 nm AuNPs indicates their potential therapeutic value in inflammatory related disorders.

In the case of treatment of rheumatoid arthritis with gold compounds, the gold has been shown to be primarily excreted in the urine and faeces [51], [52]. Nephrotoxicity has been demonstrated by minor and transient proteinuria in gold treated patients [53], [54]. This may progress to glomerulonephritis with nephritic syndrome in a small number of patients, however it is usually a short-term disorder with full recovery [55], [56]. In this study, AuNPs were not seen in the kidney albeit reduced kidney weight was observed; however, it also has to be noted that kidney weights in AuNP group were similar to those in the Saline group, indicating this may be a natural variation. It has been reported that particles larger than 10 nm should be filtered by the glomerulus [57] but the absence of AuNP within the kidney structure in the present instance suggests that this did not happen. Urine screening did not show any trace of blood or protein, while histological examination of the kidney tissue itself did not reveal any structural damage. Therefore, it can be concluded there is unlikelihood of kidney toxicity. Longer term studies are needed to determine route of excretion of the particles.

Liver is a common route for chemicals and toxins to be eliminated from the body via the faeces. In a previous *in vivo* study, following intravenous administration, AuNPs (∼32 nm diameter) tended to accumulate in the liver and were excreted via the bile [40], [58]_ENREF_33. In the current study, AuNPs appeared in macrophage-like cells within the sinuses of liver, in addition to their distribution being scattered between hepatocytes. This suggests that AuNPs of >21 nm may be eliminated mainly by the liver. Mild acute hepatic inflammatory response and apoptosis have been observed in previous studies upon AuNP administration [59]. However, such response might still be specific to AuNP size and surface modification. The main molecular mechanism by which nanoparticles produce toxicity has been hypothesized to be from an increase in oxidative stress [60]. In this study, plasma ALT was measured as an indicator of liver cell damage. In the short term, liver cells were not damaged by the presence of AuNPs as assessed by ALT.

AuNPs can be prepared as a therapeutic agent for both injection and oral administration. As such, AuNPs can be administered via subcutaneous, intravenous and intraperitoneal routes, orally ingested or inhaled. Injection is more effective than oral intake, as only 1% of injectable gold is absorbed when given orally compared to 100% when given intramuscularly [61]. In the current study, AuNPs were administered via intraperitoneal route. AuNPs were rapidly distributed in the abdominal fat tissue and liver and found in the blood vessels of these two tissues. Injection can also avoid the common gastrointestinal complications associated with oral administration of gold complexes [62]. Size is a critical factor in the *in vivo* distribution of AuNPs. It was reported that AuNPs sized greater than 20 nm do not cross the blood-brain barrier.^48^ Indeed, in the current mouse study, no AuNPs were observed in the brain sessions at any of the studied time points. AuNPs were mostly found in the abdominal fat pads and liver after i.p. injection. Consistent with the LA-ICPMS results, AuNPs were located predominantly within the periphery of adipose tissue sections with rare clusters seen deeper into the adipose tissue, AuNPs thus tended to accumulate at the outer edge of adipose tissue sections. Over time, AuNPs migrated further into the adipose tissue, and accumulated within the connective tissue between adipocytes, where ATMs are usually located. The location of AuNPs in adipose tissue appeared to vary with time; however there did not appear to be any obvious decrease in the number of AuNPs over time. This observation indicates a slow elimination of AuNPs within the adipose tissue. This could be due to their high binding capacity to albumin. Studies have shown that within the circulation, administered AuNPs could remain for several months after being bound to plasma albumin and/or globulin [63]. This characteristic could enable a long interval of repeat administration in any future applications.

The findings in the current study envisages the first application of unmodified spherical 21 nm AuNPs in the treatment of obesity-related metabolic disorders. Future study is also needed to confirm the long-term organ safety and their application in rodent models of inflammatory related disorders.
